# Time-scale of minor HIV-1 complex circulating recombinant forms from Central and West Africa

**DOI:** 10.1186/s12862-016-0824-8

**Published:** 2016-11-16

**Authors:** Edson Delatorre, Gonzalo Bello

**Affiliations:** Laboratório de AIDS e Imunologia Molecular, Instituto Oswaldo Cruz – FIOCRUZ, Av. Brasil 4365, 21040-360 Rio de Janeiro, RJ Brazil

**Keywords:** HIV-1, Complex CRFs, Africa, Phylodynamics, Phylogeography

## Abstract

**Background:**

Several HIV-1 circulating recombinant forms with a complex mosaic structure (CRFs_cpx) circulate in central and western African regions. Here we reconstruct the evolutionary history of some of these complex CRFs (09_cpx, 11_cpx, 13_cpx and 45_cpx) and further investigate the dissemination dynamic of the CRF11_cpx clade by using a Bayesian coalescent-based method.

**Results:**

The analysis of two HIV-1 datasets comprising 181 *pol* (36 CRF09_cpx, 116 CRF11_cpx, 20 CRF13_cpx and 9 CRF45_cpx) and 125 *env* (12 CRF09_cpx, 67 CRF11_cpx, 17 CRF13_cpx and 29 CRF45_cpx) sequences pointed to quite consistent onset dates for CRF09_cpx (~1966: 1958–1979), CRF11_cpx (~1957: 1950–1966) and CRF13_cpx (~1965: 1958–1973) clades; while some divergence was found for the estimated date of origin of CRF45_cpx clade [*pol* = 1970 (1964–1976); *env* = 1960 (1952–1969)]. Phylogeographic reconstructions indicate that the HIV-1 CRF11_cpx clade most probably emerged in Cameroon and from there it was first disseminated to the Central Africa Republic and Chad in the early 1970s and to other central and western African countries from the early 1980s onwards. Demographic reconstructions suggest that the CRF11_cpx epidemic grew between 1960 and 1990 with a median exponential growth rate of 0.27 year^−1^, and stabilized after.

**Conclusions:**

These results reveal that HIV-1 CRFs_cpx clades have been circulating in Central Africa for a period comparable to other much more prevalent HIV-1 group M lineages. Cameroon was probably the epicenter of dissemination of the CRF11_cpx clade that seems to have experienced a long epidemic growth phase before stabilization. The epidemic growth of the CRF11_cpx clade was roughly comparable to other HIV-1 group M lineages circulating in Central Africa.

**Electronic supplementary material:**

The online version of this article (doi:10.1186/s12862-016-0824-8) contains supplementary material, which is available to authorized users.

## Background

The HIV-1 group M epidemic started to spread in Kinshasa (Democratic Republic of Congo - DRC) long before the identification of the first AIDS cases in western countries [1, 2]. While still confined to Central Africa, the HIV-1 group M diversified into several lineages known nowadays as subtypes (named A-D, F-H, J and K) and inter-subtype recombinant forms [1]. Recombinants between HIV-1 subtypes are designated as circulating recombinant forms (CRFs; 79 described to date [Los Alamos HIV database; http://www.hiv.lanl.gov/]) if the variant is found in at least three individuals with no direct epidemiological linkage, and, if the CRF is composed by sequences originating from more than two subtypes, it is classified as complex [3]. The CRFs are increasingly becoming relevant to the HIV-1 epidemic, since the global proportion of all CRFs combined increased from 11.5% in 2000–2003 to 16% in 2004–2007 [4].

Some of the complex CRFs (including CRF04_cpx [5], CRF06_cpx [6], CRF09_cpx [7], CRF11_cpx [8], CRF13_cpx [9], CRF18_cpx [10], CRF25_cpx [11], CRF27_cpx [12], CRF37_cpx [13], CRF45_cpx [14] and CRF49_cpx [15]) carry fragments of rare subtypes (e.g., subtypes H, J and K) and divergent unclassified (U) lineages likely derived from parental strains that may predate the current subtypes [16]. Some of these complex CRFs are widely dispersed in a given African region and reaching particularly high prevalence (40–50%) in certain countries, such as the CRF06_cpx in Burkina Faso [17] and the CRF11_cpx in the Central African Republic [18]. Others complex CRFs circulate at a very low prevalence (<5%) throughout several countries from West (CRF09_cpx and CRF49_cpx) [15, 17, 19–21] and Central (CRF13_cpx, CRF45_cpx) [22–25] Africa. The remaining of those complex CRFs were sporadically detected in Africa, but have successfully disseminated to other locations, as the CRF04_cpx in Greece and Cyprus [26, 27] and the CRF18_cpx in Cuba [10].

Information about the time-scale, migration routes and population dynamics of those complex CRFs (CRFs_cpx) are scarce. Previous studies conducted by our group support that the CRF06_cpx epidemic in West Africa probably originated from the regional dissemination of a single founder strain introduced in Burkina Faso around the late 1970s [28], while the CRF18_cpx epidemic in Cuba probably resulted from the local expansion of a single founder strain introduced in the country at the early 1990s [29]. Although these estimates supports a relative recent origin for CRF06_cpx and CRF18_cpx epidemics in West Africa and Cuba, these and other CRFs_cpx carrying fragments of rare subtypes and U lineages probably arose in Central Africa several years earlier. The precise onset dates of the CRFs_cpx clades at the epicenter, however, remain largely unknown.

In the present study, we reconstructed the time-scale of the CRFs 09_cpx, 11_cpx, 13_cpx and 45_cpx as well as the spatial and demographic dissemination dynamics of the CRF11_cpx, by using two comprehensive data sets of HIV-1 *pol* (*n* = 181) and *env* (*n* = 125) sequences sampled in Central and West Africa over a period of 27 years.

## Methods

### HIV-1 CRFs_cpx sequences datasets

All CRF09_cpx, CRF11_cpx, CRF13_cpx and CRF45_cpx (CRFs09/11/13/45_cpx) sequences with information about country of origin and sampling date were retrieved from the Los Alamos HIV Sequence Database (Los Alamos HIVdb, www.hiv.lanl.gov). The sequences covered the entire protease and partial reverse transcriptase (PR/RT) regions of *pol* gene corresponding to HXB2 coordinates 2253 to 3272 (CRFs_cpx *pol* dataset), and the V3 region of the *env*-*gp120* gene corresponding to HXB2 coordinates 7041 to 7345 (CRFs_cpx *env* dataset). All sequences of the CRFs09/11/13/45_cpx’s parental subtypes (subtypes J and K for *pol* fragment and subtypes A, A1, A2 for *env* fragments) from Central and West African countries were also retrieved from Los Alamos HIVdb and included in the final datasets. Sequences were aligned using CLUSTAL X v.2 program [30], followed by manual editing.

### Genetic classification of HIV-1 CRFs_cpx

The subtype classification of all *pol* and *env* sequences here included was initially verified with REGA HIV subtyping tool v.3 [31] and COMET v.2 [32] and further confirmed by Maximum Likelihood (ML) phylogenetic and bootscanning analysis. The ML tree was inferred with PhyML program [33] using an online web server [34] under the GTR + I + Γ_4_ nucleotide substitution model selected using the jModeltest v.2 program, and the SPR branch-swapping algorithm for heuristic tree search. The consistency of the tree topology was estimated with approximate likelihood-ratio test [35] based on a Shimodaira-Hasegawa-like procedure (SH-*a*LRT). All CRFs_cpx sequences were inspected to verify if their mosaic profile were the expected according to the published CRF breakpoint locations in the Los Alamos HIVdb database (Additional file 1: Figure S1) by bootscanning analysis using Simplot software v.3.5.1 [36]. Bootstrap values supporting branching of query and reference sequences were determined by Neighbor-Joining trees constructed using the Kimura two-parameter model based on 100 re-samplings, with a 300 bp sliding window moving in steps of 10 bases.

### Evolutionary analyses

The phylogenetic tree, evolutionary rate (μ, nucleotide substitutions per site per year, subst./site/year) and the age of the most recent common ancestor (T_MRCA_, years) of HIV-1 CRFs09/11/13/45_cpx epidemics circulating in Central and West African regions were jointly estimated using a Bayesian Markov Chain Monte Carlo (MCMC) approach implemented in BEAST v1.8.0 [37, 38] along with BEAGLE v2.1 library to perform parallelization [39]. Analyzes were performed using the GTR + I + Γ_4_ nucleotide substitution model, an uncorrelated lognormal relaxed molecular clock model [40] with informative substitution rate priors for the *pol* (1.5 × 10^−3^–3.0 × 10^−3^ subst./site/year) and *env* (4 × 10^−3^–8 × 10^−3^ subst./site/year) [41] genomic regions, and a Bayesian Skyline coalescent tree prior [42]. MCMC chains were run for 10^8^generations and adequate chain mixing was checked, after excluding an initial 10% burn-in for each run, by calculating the effective sample size (ESS) using TRACER v1.6 program [43]. Maximum clade credibility (MCC) trees were summarized from the posterior distribution of trees with TreeAnnotator and visualized with FigTree v1.4 [44].

### Phylogeographic and demographic analyses

The spatiotemporal and demographic dynamics of dissemination of the HIV-1 CRF11_cpx clade were reconstructed using BEAST v1.8.0 as previously described. Migration events throughout the *pol* and *env* phylogenetic history were reconstructed by applying a reversible discrete Bayesian phylogeographic model [45] and a continuous-time Markov chain rate reference prior [46] and latter summarized using the SPREAD v.1.0.6 application [47]. The effective population size through time was initially estimated using a Bayesian Skyline coalescent model [42] and estimates of the population growth rate were subsequently obtained using different parametric models (logistic, exponential and expansion) [38]. The fittest model to the demographic signal contained in CRF11_cpx *pol* and *env* dataset was chosen after model comparison using the log marginal likelihood estimation based on path sampling (PS) and stepping-stone sampling (SS) methods [48]. MCMC chains were run for 10^8^ generations and adequate chain mixing was checked as previously described. Graphical representations of the effective number of infections through time were generated by programs TRACER v1.6 [43] and GraphPad Prism 6 (GraphPad Software).

## Results

### Selection of HIV-1 CRFs_cpx *pol* and *env* sequences

Most CRFs_cpx-like *pol* (99.4%) and *env* (98.5%) sequences obtained from Los Alamos HIVdb were correctly genotyped since displayed the same mosaic structures (Additional file 1: Figure S1) and branched in highly supported monophyletic clades (Additional file 2: Figure S2 and Additional file 3: Figure S3) with corresponding CRFs_cpx reference sequences. These analyses also identified 67 sequences erroneously classified and misannotated in the Los Alamos HIVdb (Additional file 4: Table S1). Two *pol* sequences incorrectly annotated as subtype J and CRF11_cpx were reclassified as CRF11_cpx and CRF13_cpx, respectively (Additional file 2: Figure S2). One CRF09_cpx *env* sequence branched within the CRF11_cpx clade, while 57 subtype A/A1/A2 *env* sequences branched within the CRFs09/11/13/45_cpx clades radiations and were thus reclassified accordingly (Additional file 3: Figure S3). This approach resulted in two final datasets composed by 181 CRFs_cpx-like *pol* sequences and 125 CRFs_cpx-like *env* sequences, sampled between 1984 and 2011 from 16 countries of Central and West Africa (Additional file 5: Table S2 and Additional file 6: Table S3) that were used for the subsequent analyses.

The ML *env* phylogenetic tree also allowed drawing inferences about the evolutionary origin of the parental viruses that originated the CRFs_cpx, once it was reconstructed from a common subtype A genomic segment. The subtype A segments of CRF11_cpx and CRF13_cpx lineages form a highly supported cluster (SH-*a*LRT = 0.96) within the subtype A/A1 radiation that also comprised 16 basal sequences originated almost exclusively from Central Africa (DRC/Congo). In contrast, the subtype A segments of CRF09_cpx and CRF45_cpx lineages branched outside the subtype A/A1 radiation as very early divergent lineages. Two sequences from the DRC branched basally to the CRF45_cpx clade (SH-*a*LRT = 0.85), whereas no basal sequences to the CRF09_cpx clade were identified. The A/A1 *env* sequences from Central Africa that clustered basally to the CRFs_cpx clades with high support (SH-*a*LRT > 0.90) were combined with the CRFs09/11/13/45_cpx dataset to aid Bayesian evolutionary and temporal analyses.

### Time-scale of HIV-1 CRFs_cpx clades

**Fig. 1:**
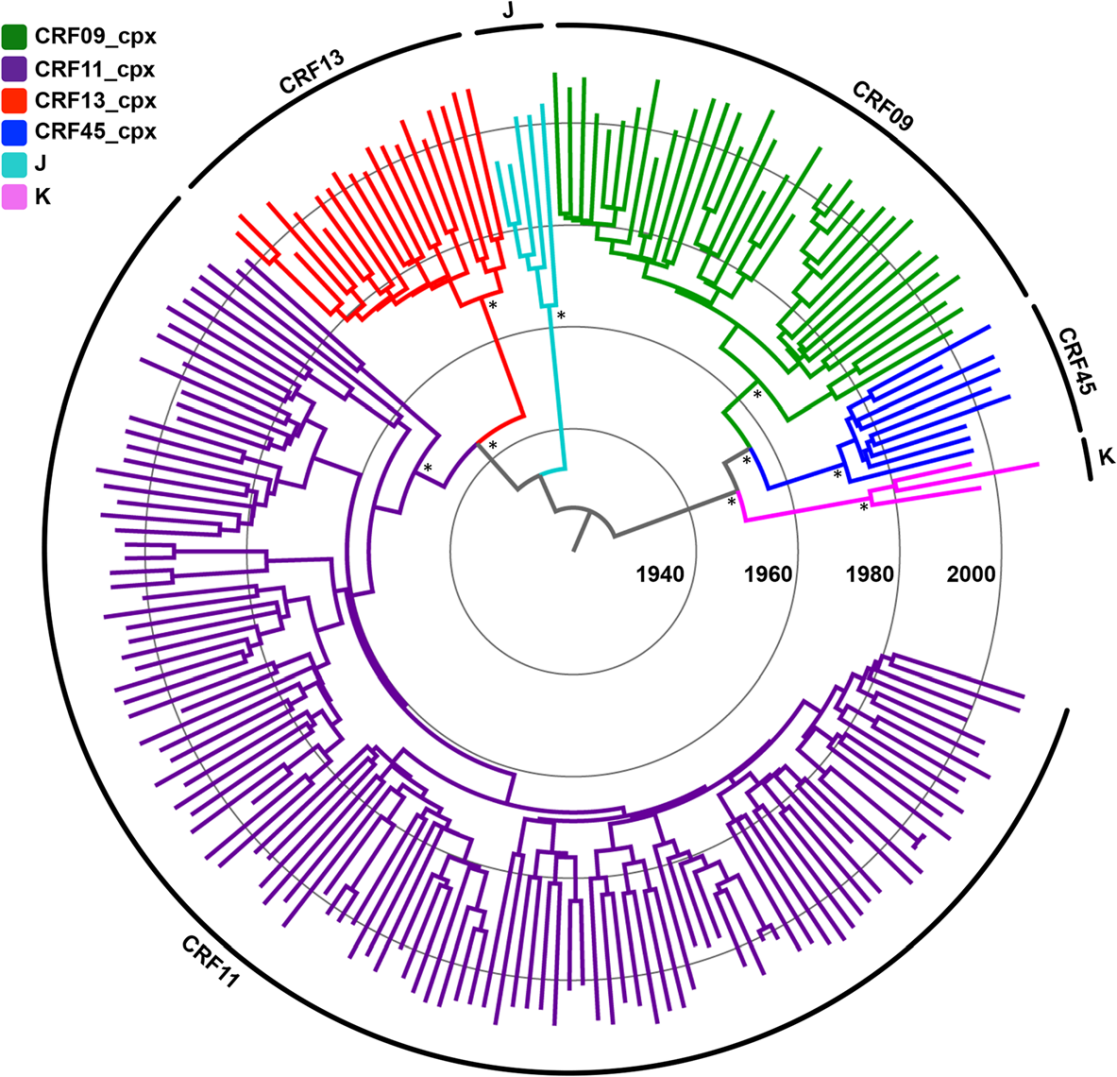
Time-scaled Bayesian MCC tree of the HIV-1 CRFs09/11/13/45_cpx *pol* gene fragment. Branch color indicates the subtype classification obtained in this study, according to the legend in top left. The external circular segments highlight the position of each specific clade as indicated at the line. Asterisks point to key nodes with a high (> 0.90) *PP* support. Branch lengths are drawn to scale with the concentric circles indicating years. The tree was automatically rooted under the assumption of a relaxed molecular clock

**Fig. 2:**
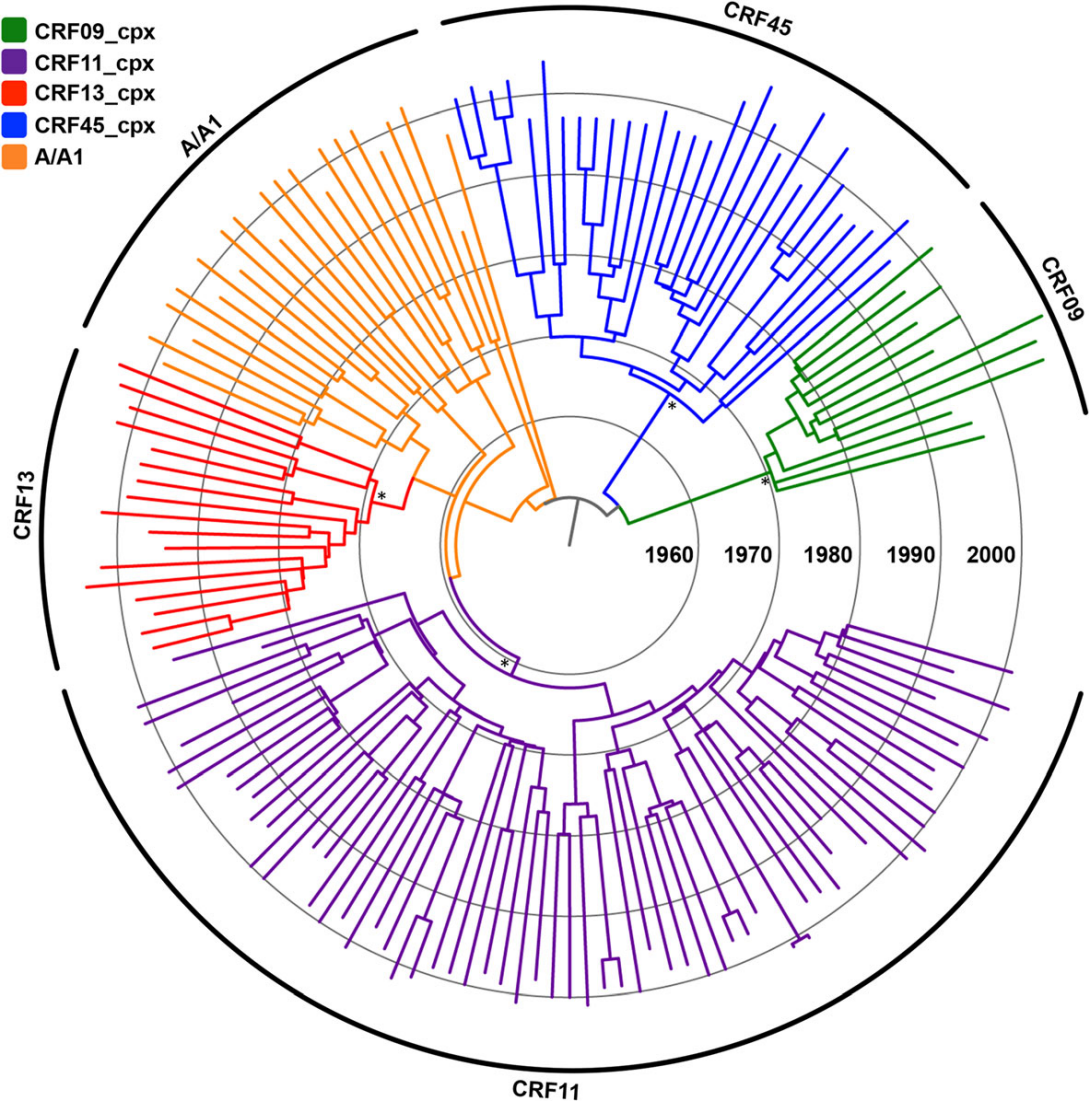
Time-scaled Bayesian MCC tree of the HIV-1 CRFs09/11/13/45_cpx *env* gene fragment. Branch color indicates the subtype classification obtained in this study, according to the legend in top left. The external circular segments highlight the position of each specific clade as indicated at the line. Asterisks point to key nodes with a high (> 0.90) *PP* support. Branch lengths are drawn to scale with the concentric circles indicating years. The tree was automatically rooted under the assumption of a relaxed molecular clock

Bayesian analyses of both *pol* and *env* datasets confirmed that all sequences from a given CRFs_cpx formed highly supported monophyletic clades (posterior probability, PP > 0.90) (Figs. 1 and 2) with an overall weak geographic structure (Additional file 7: Figure S4). The median evolutionary rate calculated under a relaxed molecular clock model was 1.6 × 10^−3^ subst./site/year for *pol* gene and 4.3 × 10^−3^subst./site/year for *env* gene. The coefficient of rate variation for both genes was significantly higher than zero (Table 1), thus supporting the use of a relaxed molecular clock model. The median T_MRCA_ obtained from both HIV-1 datasets point to quite consistent onset dates for the CRF09_cpx (*pol* = 1968, *env* = 1965), CRF11_cpx (*pol* = 1958, *env* = 1957) and CRF13_cpx (*pol* = 1966, *env* = 1964) clades (Table 1). A slightly younger median T_MRCA_ for the CRF45_cpx clade was obtained for *pol* (1970) than for *env* (1960) datasets. This can be probably attributed to the much smaller sample size of the CRF45_cpx-like *pol* dataset (*n* = 9) when compared to the *env* dataset (*n* = 29).

**Table 1:** Bayesian estimates of evolutionary parameters of the HIV-1 CRFs_cpx clades

Gene	*μ* ^a^ (subst./site/year)	Coeficient of rate variation^a^	CRF	*N* (sampling years)	Tmrca^a^
*pol*	1.6 × 10^−3^ (1.5 × 10^−3^–1.8 × 10^−3^)	0.28 (0.23–0.33)	CRF09_cpx	36 (1995–2011)	1968 (1961–1973)
			CRF11_cpx	116 (1995–2011)	1958 (1950–1966)
			CRF13_cpx	20 (1996–2009)	1966 (1959–1973)
			CRF45_cpx	9 (1997–2009)	1970 (1964–1976)
*env*	4.3 × 10^−3^ (4.0 × 10^−3^–5.1 × 10^−3^)	0.26 (0.20–0.33)	CRF09_cpx	12 (1996–2009)	1965 (1958–1979)
			CRF11_cpx	67 (1984–2002)	1957 (1950–1965)
			CRF13_cpx	17 (1994–2004)	1964 (1958–1971)
			CRF45_cpx	29 (1997–2006)	1960 (1952–1969)

^a^The 95% HPD interval is displayed in parentheses

### Spatial and demographic dissemination dynamics of the CRF11_cpx clade

Phylogeographic and demographic reconstructions were only performed for the CRF11_cpx clade, since it was the only one that comprised a number of *pol* and *env* sequences large enough (*n* > 30) to provide accurate estimates.

**Fig. 3:**
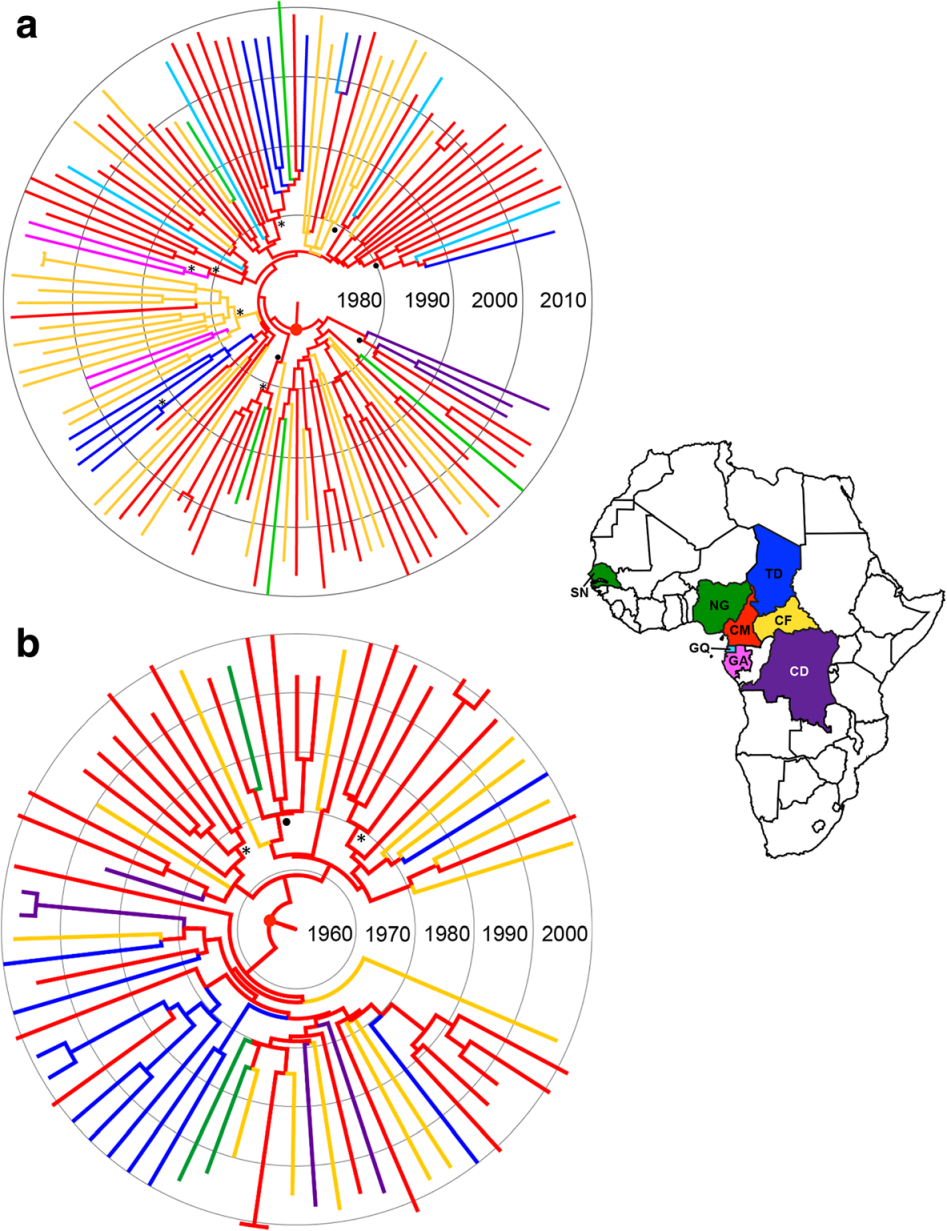
Time-scaled Bayesian MCC phylogeographic trees of HIV-1 CRF11_cpx *pol* (**a**) and *env* (**b**) datasets. The color of each branch represents the most probable location origin according to the map given in the figure. Nodes with a relative-high (*PP* > 0.80 and < 0.90) and high support (*PP* > 0.90) are marked with black dots and asterisks, respectively. The red dots represent Cameroon as the ancestral root state with location posterior probabilities of 0.99 and 0.98 for *pol* and *env* datasets, respectively. Branch lengths are drawn to scale with the concentric circles indicating years. Localities represented are: DRC (CD), Central African Republic (CF), Cameroon (CM), Gabon (GA), Equatorial Guinea (GQ), Chad (TD) and West African countries (WA)

**Fig. 4:**
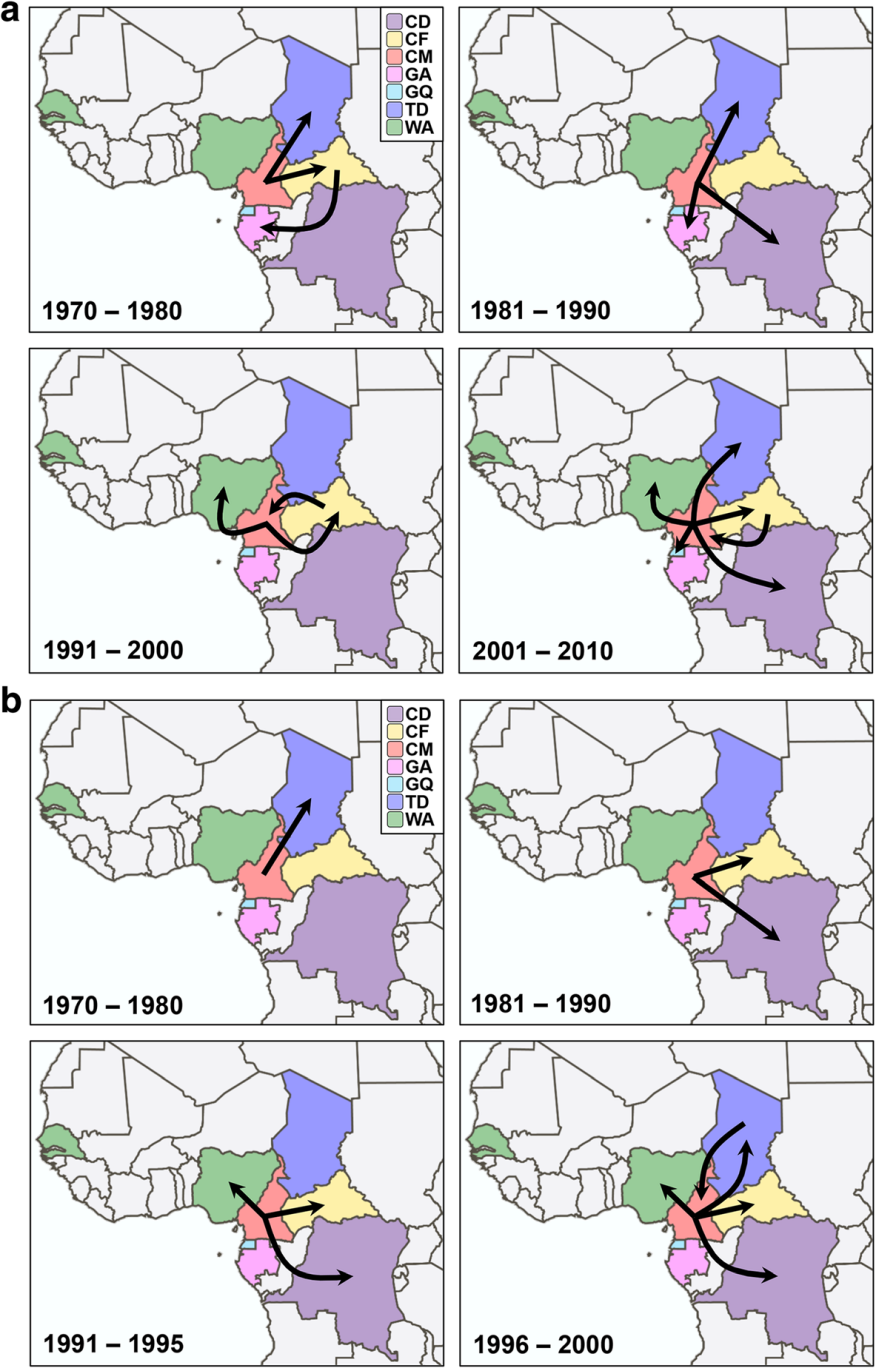
Spatiotemporal dispersion of the HIV-1 CRF11_cpx in Central and West Africa. Viral migration events were estimated for *pol* (**a**) and *env* (**b**) fragments. Arrows between locations represent branches in the Bayesian MCC tree along which location transitions occurred. Each panel represents a time interval of locations transitions as reported. Locations are colored according to the legend. Localities codes: CD (DRC), CF (Central African Republic), CM (Cameroon), GA (Gabon), GQ (Equatorial Guinea), TD (Chad) and West African countries (WA)

The evolutionary parameters obtained from both CRF11_cpx datasets were almost identical to those estimated from the combined CRFs09/11/13/45_cpx datasets (Table 2). The patterns of viral migration across time reconstructed from both *pol* and *env* phylogenies were very similar and indicated that the CRF11_cpx clade most probably emerged in Cameroon (posterior state probability ≥ 0.98) around the early 1960s (Figs. 3 and 4). From Cameroon, the CRF11_cpx was first disseminated to Chad and the Central African Republic between 1970 and 1980, and to other neighboring Central (DRC, Equatorial Guinea and Gabon) and West (Nigeria) African countries from the early 1980s onwards. Secondary disseminations of the CRF11_cpx from the Central African Republic to Cameroon/Gabon and from Chad to Cameroon were also recovered by the *pol* and *env* datasets, respectively.

**Table 2:** Bayesian estimates of evolutionary and population dynamic parameters of the HIV-1 CRF11_cpx clade

Gene	Coalescent	*μ* (subst./site/year)	T_MRCA_	*r* (year^−1^)
*pol*	Bayesian skyline	1.6 × 10^−3^ (1.5 × 10^−3^–2.0 × 10^−3^)	1961 (1953–1970)	-
	Logistic growth	1.6 × 10^−3^ (1.5 × 10^−3^–1.9 × 10^−3^)	1958 (1951–1967)	0.27 (0.21–0.35)
*env*	Bayesian skyline	4.9 × 10^−3^ (4.2 × 10^−3^ – 5.8 × 10^−3^)	1957 (1954–1961)	-
	Logistic growth	4.9 × 10^−3^ (4.2 × 10^−3^ – 5.7 × 10^−3^)	1957 (1953–1961)	0.28 (0.21–0.35)

The 95% HPD interval is displayed in parentheses

**Fig. 5:**
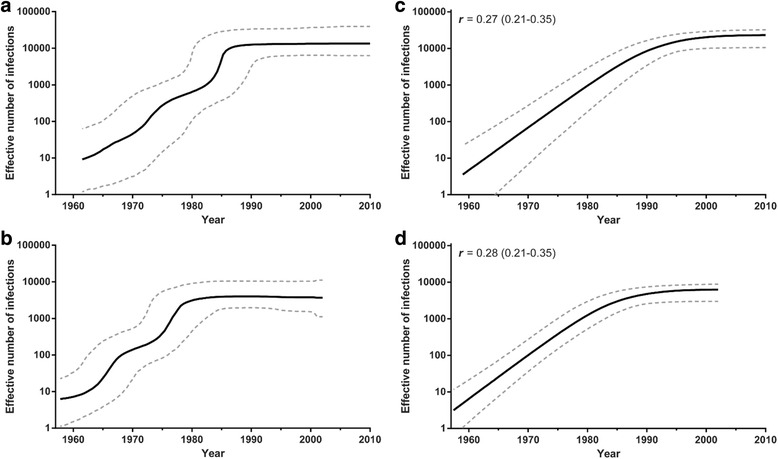
Demographic history of the HIV-1 CRF11_cpx epidemic. Non-parametric estimates of effective number of infections through time of the HIV-1 CRF11_cpx epidemic in *pol* (**a**) and *env* (**b**) datasets are represented by Bayesian skyline plots. Demographic history of HIV-1 CRF11_cpx epidemic based on *pol* (**c**) and *env* (**d**) datasets were reconstructed using a logistic growth coalescent model. Median estimate of the effective number of infections (solid line) and 95% confidence limits of the estimate (dashes lines) are shown. Vertical axes indicate the estimated effective number of infections and were represented on logarithmic scale. Time scale is in calendar years

The changes in the effective population size (*Ne*) of the CRF11_cpx clade over time estimated from both *pol* and *env* datasets were also very similar. The Bayesian skyline plot (BSP) coalescent analysis indicated that the CRF11_cpx clade experienced an initial phase of exponential growth, followed by a decline in growth rate from the mid-1980s (Fig. 5 and Table 2). Consistent with this result, the logistic growth model was pointed as the best-fit parametric demographic model (log Bayes Factor > 20) by both PS and SS methods (Additional file 8: Table S4) and then it was used to estimate the epidemic growth rate of the CRF11_cpx epidemic in Central Africa. According to the logistic growth coalescent model, the CRF11_cpx expanded between 1960 and 1990 with a median growth rate of 0.27 year^−1^ (*pol*) and 0.28 year^−1^ (*env*) (Fig. 5 and Table 2).

## Discussion

The pervasive recombination of the HIV-1 at the early stages of the group M epidemic generated a large array of complex CRFs at the epicenter in Central Africa that usually circulates at low prevalence [5–15]. In this study, we compile a quite large number of HIV-1 *pol* and *env* sequences from four complex recombinants (CRF09_cpx, CRF11_cpx, CRF13_cpx and CRF45_cpx) displaying unique mosaic structures in *pol* and a common subtype A fragment in *env* and estimate their onset dates. Most CRFs_cpx-like *pol* sequences used here were correctly annotated in the Los Alamos HIVdb. A significant fraction (46%) of the CRFs_cpx-like *env* sequences here recovered, by contrast, corresponded to West and Central African sequences originally classified as subtype A/A1/A2, but that clearly branched within the CRFs_cpx radiations and should be thus reclassified.

The inspection of the *env* ML and Bayesian phylogenetic trees revealed that the subtype A *env* segments that originated the ancestors of CRF11_cpx and CRF13_cpx seems to have derived from a common lineage that currently circulates in DRC and Congo. Other interesting observation was that while subtype A *env* segment of CRF11_cpx and CRF13_cpx fall within the subtype A/A1 radiation, the subtype A *env* segments of CRF09_cpx and CRF45_cpx branched as early divergent lineages basally to the root of subtype A/A1. This observation is consistent with the notion that CRF09_cpx and CRF45_cpx likely originated from viruses that diverged close to (or even before) the time of the HIV-1 subtype A progenitor [16]. Our results also points that some of these early divergent lineages are still circulating in the DRC, once two HIV-1 subtype A-like sequences from that country branched basally to the CRF45_cpx clade. The contributions of these lineages to the genesis of some CRFs indicate that they had epidemiological relevance during the early stages of the HIV-1 group M epidemic [16, 49].

It was suggested that the low frequencies of many ancient HIV-1 divergent lineages in the human population was caused by its absence during the initial migratory wave of variants that triggered the global epidemic [16]. Similarly, the overall low prevalence of the CRFs_cpx lineages carrying fragments of those ancient may reflect a later emergence of these variants when compared to more prevalent HIV-1 subtypes and CRFs. The evolutionary analyses performed here, however, support that complex CRFs probably started to circulate in Central Africa between the late 1950s and the late 1960s, which coincides with the estimated onset date of several prevalent HIV-1 group M clades including: subtype A1 (T_MRCA_ ~ 1955) [41], subtype C (T_MRCA_ ~ 1955–1965) [41, 50, 51], subtype F1 (T_MRCA_ ~ 1960–1970) [52, 53], subtype G (T_MRCA_ ~ 1965–1970) [41, 54], the CRF01_AE (T_MRCA_ ~ 1970–1975) [41, 55, 56], and the CRF02_AG (T_MRCA_ ~ 1965–1975) [41, 57, 58]. The estimated T_MRCA_ also overlaps with a period of demographic transition of the HIV-1 group M in the DRC around 1960 (95% HPD: 1952–1968), from an early phase of relatively slow exponential growth to a second phase of faster exponential growth [2]. Thus, the early establishment of most HIV-1 group M subtypes and CRFs in the DRC was probably shaped by the same factors and occurred at around the same time, despite significant disparities in their final epidemic outcomes.

Alternatively, the current low prevalence of many ancient HIV-1 divergent lineages and CRFs_cpx lineages may reveals a lower transmissibility of those variants when compared with the globally circulating HIV-1 clades [16]. Some evidences, however, also argued against this hypothesis. First, the CRF06_cpx and CRF11_cpx clades comprise a large fraction (40–50%) of HIV-1 infections in Burkina Faso [17] and the Central African Republic [18], respectively, and the CRF18_cpx was successfully disseminated in Cuba [10]. Second, coalescent estimations of the exponential growth rates of the CRF06_cpx (~0.8 year^−1^) and CRF18_cpx (~0.6 year^−1^) clades in West Africa [28] and Cuba [29], respectively, were similar to that estimated for highly prevalent HIV-1 lineages including: subtype B in Western countries (~0.5–1.5 year^−1^) [59–62], subtype C in Brazil (~0.5–0.9 year^−1^) [63, 64], and subtype G (~0.7–1.0 year^−1^) and CRF02_AG (~0.6 year^−1^) in West Africa [54, 58]. These observations demonstrate that, in specific settings, the CRFs_cpx clades were able to seed large epidemics and to spread at rates comparable to the most prevalent HIV-1 group M clades.

Our demographic reconstructions also indicate that the epidemic growth rate seems to vary for different CRFs_cpx. According to our estimations, the CRF11_cpx expanded in Central Africa between 1960 and 1990 with a median growth rate of ~0.3 year^−1^ (95% HPD: 0.2–0.4 year^−1^), a value significantly lower than that estimated for the CRF06_cpx and CRF18_cpx epidemics in West Africa and Cuba, respectively. The epidemic growth rate of the CRF11_cpx, however, was comparable to that estimated for some subtype G (0.3–0.6 year^−1^) and CRF02_AG (0.3–0.5year^−1^) clades circulating in Cameroon [54, 58] and to that estimated for the HIV-1 group M (0.2–0.3 year^−1^) in the DRC during 1960–1990 [2]. We propose that differences in epidemic growth rates across HIV-1 African lineages most probably resulted from ecological determinants, although differences in viral transmissibility properties might be also responsible for the growth rate variances in some cases [58].

Spatial accessibility has been pointed as a major driving force of HIV-1 spread within Africa, and the central African region displayed a much lower spatial connectivity than western, eastern, and southern sub-Saharan regions [65]. The CRF06_cpx clade most probably entered in Burkina Faso and from there was disseminated to other neighboring western African countries [28]. According to our phylogeographic reconstructions, the epicenter and most important hub of dissemination of the CRF11_cpx clade was Cameroon, from where the virus spread to neighboring Central African countries (the Central African Republic, Chad, Gabon and Equatorial Guinea). Thus, the dissemination of the CRF06_cpx clade took place in a geographic region much better connected than the region of dissemination of the CRF11_cpx clade, which may have contributed to the faster epidemic growth rate of the CRF06_cpx when compared to the CRF11_cpx.

## Conclusions

This study shows that HIV-1 CRFs_cpx clades were already circulating in Central Africa before the late 1960s and probably arose at around the same time than other more prevalent HIV-1 group M lineages. Cameroon was traced as the most probable epicenter of CRF11_cpx dissemination in Central Africa and the demographic history of this CRF was roughly comparable to that described for other central African HIV-1 group M lineages. These results support that the final prevalence of the different HIV-1 group M lineages circulating in human populations was mainly determined by stochastic and ecological factors, rather than by differences in the precise onset date of viral lineages. This study offers important insights toward an understanding of the epidemic potential and current dissemination pattern of some rare HIV-1 group M clades.

## Additional files

Additional file 1:
**Figure S1.** Recombination pattern of the circulant recombinant forms analyzed in this study. Box representing *pol* (HXB2: 2253–3272) and *env* (HXB2: 7041–7346) gene fragments used in this study were superimposed on the graphical illustrations of the CRFs09\11\13\45_cpx genomes based on breakpoint data available in Los Alamos HIV database and colored according to the legend at bottom. (TIF 343 kb)
Additional file 2:
**Figure S2.** Maximum likelihood phylogenetic tree based on the CRF09/11/13/45_cpx *pol* fragment sequences for HIV-1 subtype (re)classification. Branches were colored according to HIV-1 subtype classification provided by the Los Alamos HIV database and indicated at the legend. Black dots represent the reference genomes of each CRF. For visual clarity, other subtypes not directly related to the CRF09/11/13/45_cpx were collapsed into triangles. The branch support values are indicated as * (SH-*a*LRT > 0.80 and < 0.90) or ** (SH-*a*LRT > 0.90) at key nodes. Horizontal branch lengths are drawn to scale with the bar at the bottom indicating nucleotide substitutions per site. (TIF 287 kb)
Additional file 3:
**Figure S3.** Maximum likelihood phylogenetic tree based on the CRF09/11/13/45_cpx *env* fragment sequences for HIV-1 subtype (re)classification. Branches were colored according to HIV-1 subtype classification provided by the Los Alamos HIV database and indicated at the legend. Black dots represent the reference genomes of each CRF. For visual clarity, other subtypes not directly related to the CRF09/11/13/45_cpx and some clades that comprised mostly sequences of subtype A were collapsed into triangles. The branch support values are indicated as * (SH-*a*LRT > 0.80 and < 0.90) or ** (SH-*a*LRT > 0.90) at key nodes. Horizontal branch lengths are drawn to scale with the bar at the bottom indicating nucleotide substitutions per site. (TIF 490 kb)
Additional file 4:
**Table S1.** Sequences reclassified in this study. (PDF 75 kb)
Additional file 5:
**Table S2.** HIV-1 CRFs_cpx *pol* dataset. (PDF 69 kb)
Additional file 6:
**Table S3.** HIV-1 CRFs_cpx *env* dataset. (PDF 70 kb)
Additional file 7:
**Figure S4.** Geographic distribution of the HIV-1 CRFs09/11/13/45_cpx *pol* (a) and *env* (b) gene fragments. Tips colors indicate the country of isolation of each sequence, according to the map. Country names are indicated using a two-letter code in accordance with ISO 3166. The external circular segments highlight the position of each specific clade as indicated at the line. Asterisks point to key nodes with a high (>0.90) *PP* support. Branch lengths are drawn to scale with the concentric circles indicating years. The trees were automatically rooted under the assumption of a relaxed molecular clock. (TIF 2346 kb)
Additional file 8:
**Table S4.** Best-fit demographic models for HIV-1 CRF11_cpx *pol* and *env* datasets. (PDF 64 kb)
