# Supplementary figures and images for: Time-scale of minor HIV-1 complex circulating recombinant forms from Central and West Africa
Source: BMC Evol Biol. 2016 Nov 16;16:249. doi: 10.1186/s12862-016-0824-8 (PMC5112642; doi:10.1186/s12862-016-0824-8)

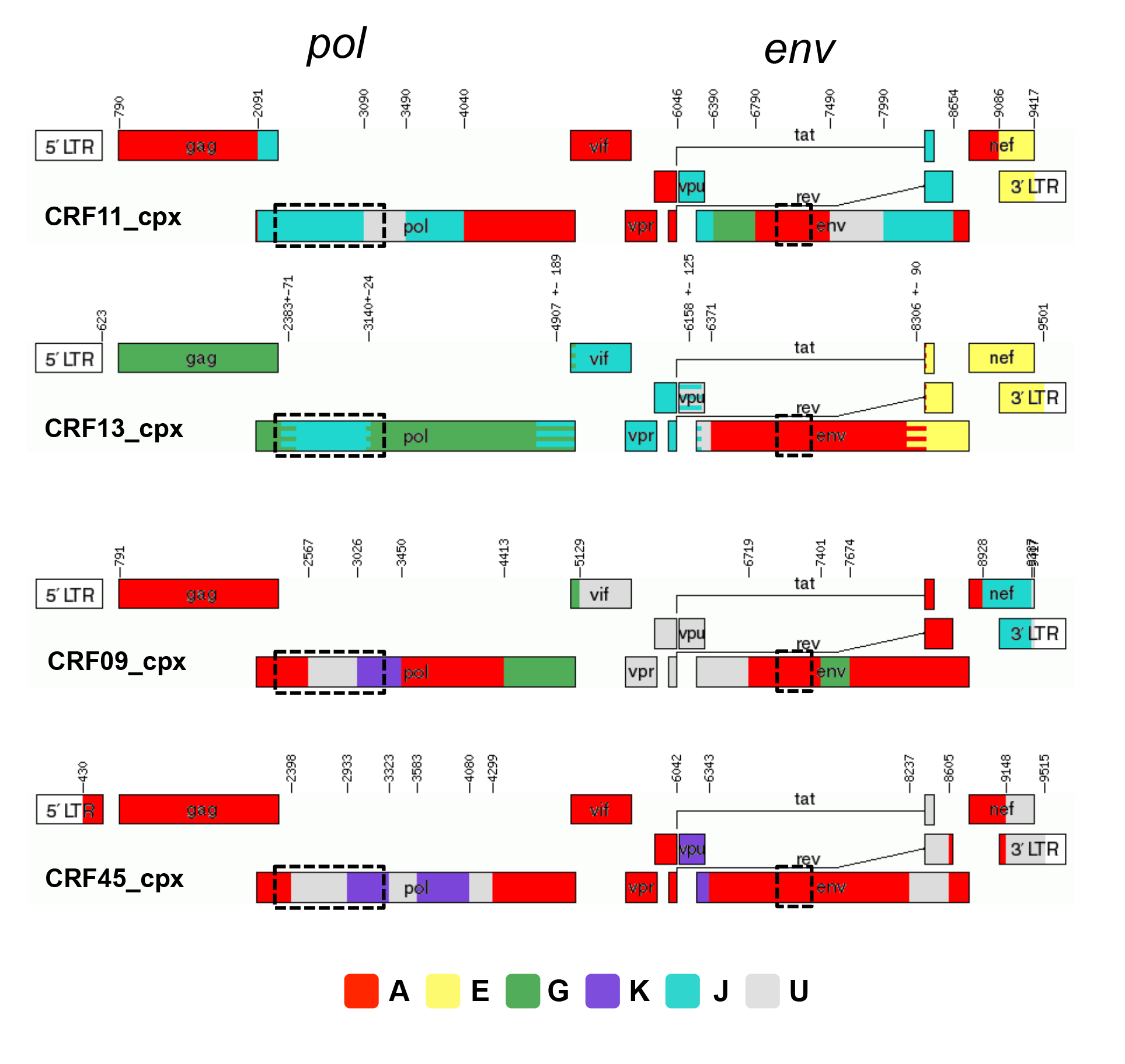

Supplement: Additional file 1: — Figure S1. Recombination pattern of the circulant recombinant forms analyzed in this study. Box representing pol (HXB2: 2253–3272) and env (HXB2: 7041–7346) gene fragments used in this study were superimposed on the graphical illustrations of the CRFs09\11\13\45_cpx genomes based on breakpoint data available in Los Alamos HIV database and colored according to the legend at bottom. (TIF 343 kb) [file 12862_2016_824_MOESM1_ESM.tif]

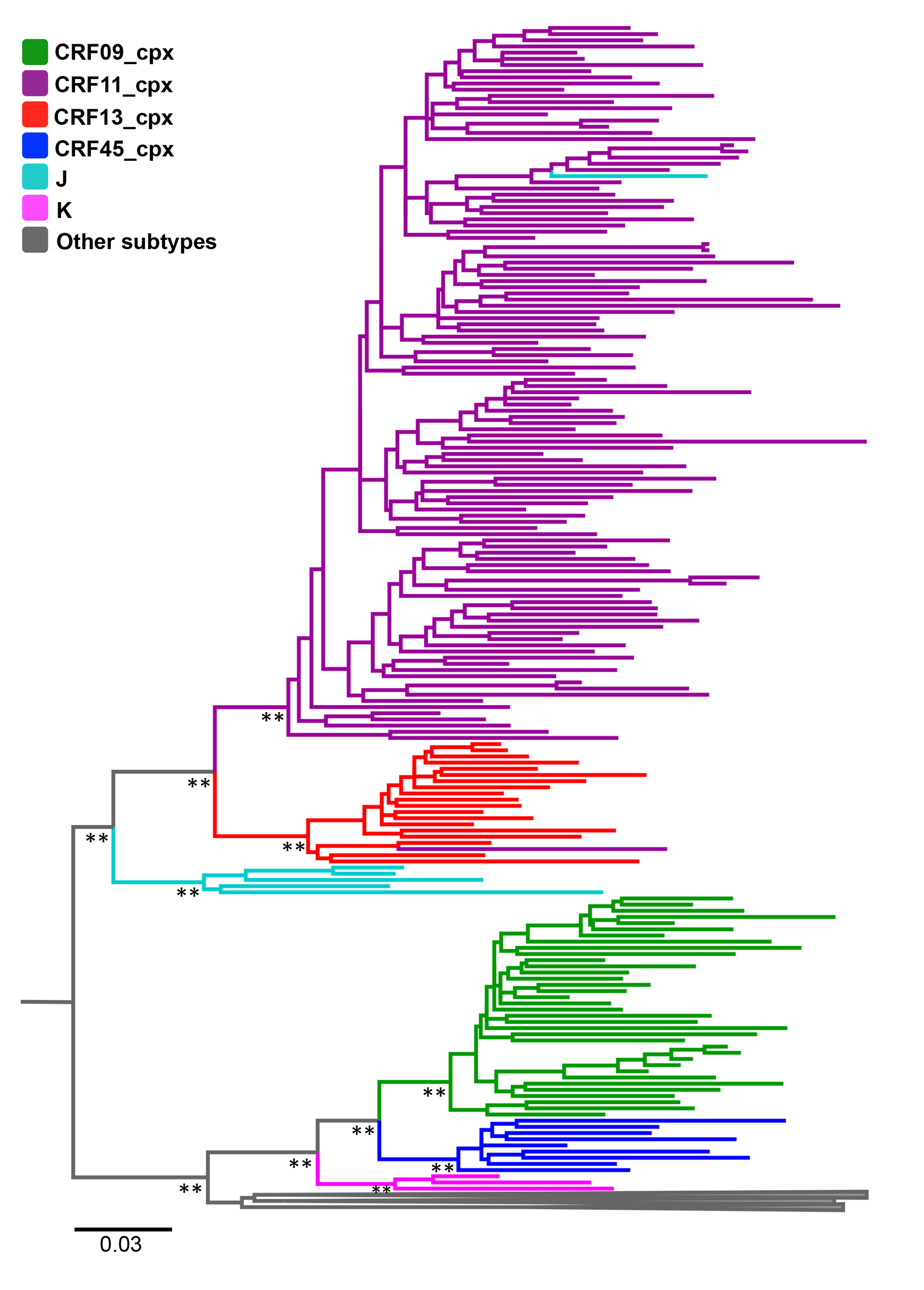

Supplement: Additional file 2: — Figure S2. Maximum likelihood phylogenetic tree based on the CRF09/11/13/45_cpx pol fragment sequences for HIV-1 subtype (re)classification. Branches were colored according to HIV-1 subtype classification provided by the Los Alamos HIV database and indicated at the legend. Black dots represent the reference genomes of each CRF. For visual clarity, other subtypes not directly related to the CRF09/11/13/45_cpx were collapsed into triangles. The branch support values are indicated as * (SH-aLRT > 0.80 and < 0.90) or ** (SH-aLRT > 0.90) at key nodes. Horizontal branch lengths are drawn to scale with the bar at the bottom indicating nucleotide substitutions per site. (TIF 287 kb) [file 12862_2016_824_MOESM2_ESM.tif]

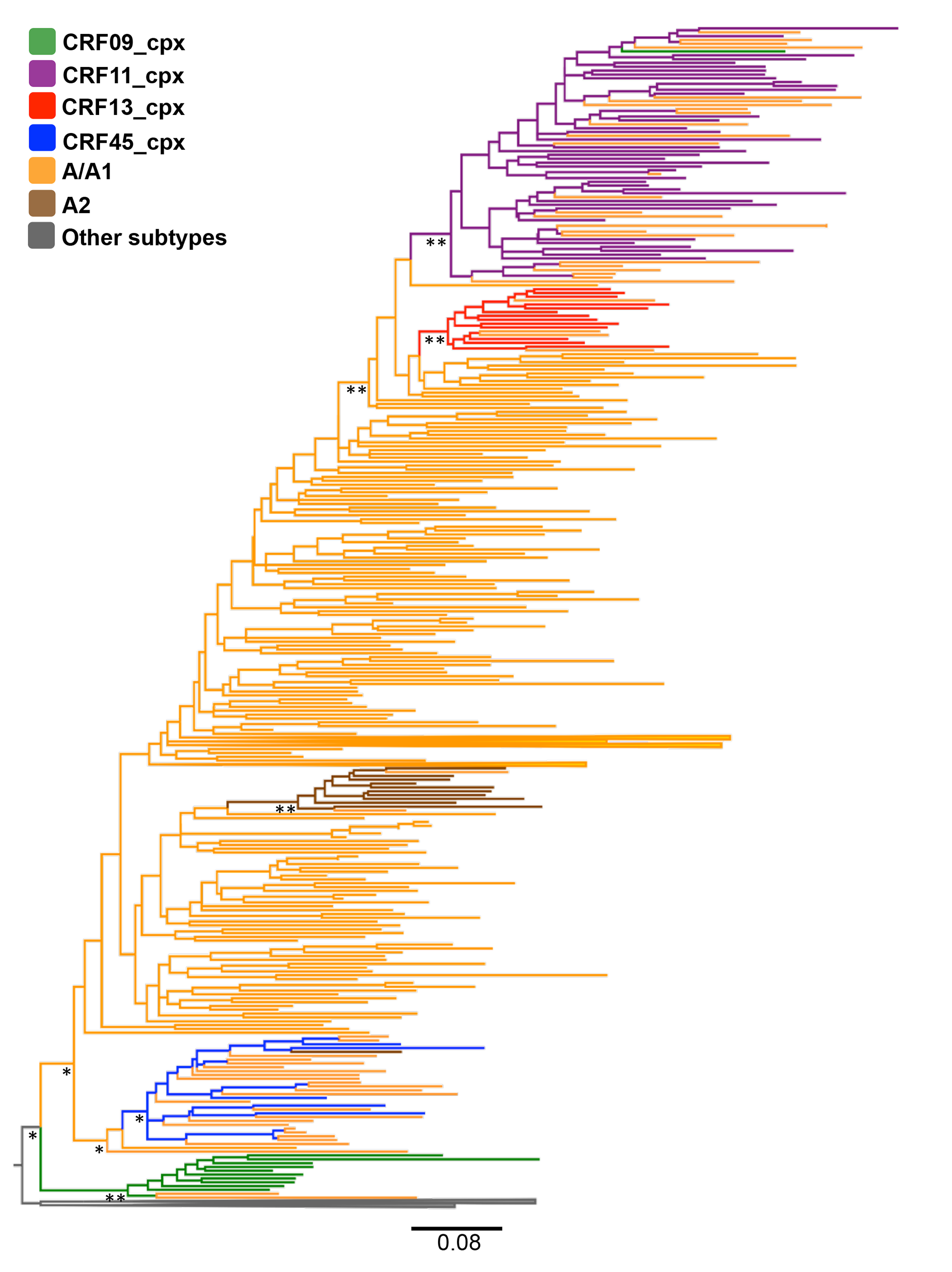

Supplement: Additional file 3: — Figure S3. Maximum likelihood phylogenetic tree based on the CRF09/11/13/45_cpx env fragment sequences for HIV-1 subtype (re)classification. Branches were colored according to HIV-1 subtype classification provided by the Los Alamos HIV database and indicated at the legend. Black dots represent the reference genomes of each CRF. For visual clarity, other subtypes not directly related to the CRF09/11/13/45_cpx and some clades that comprised mostly sequences of subtype A were collapsed into triangles. The branch support values are indicated as * (SH-aLRT > 0.80 and < 0.90) or ** (SH-aLRT > 0.90) at key nodes. Horizontal branch lengths are drawn to scale with the bar at the bottom indicating nucleotide substitutions per site. (TIF 490 kb) [file 12862_2016_824_MOESM3_ESM.tif]

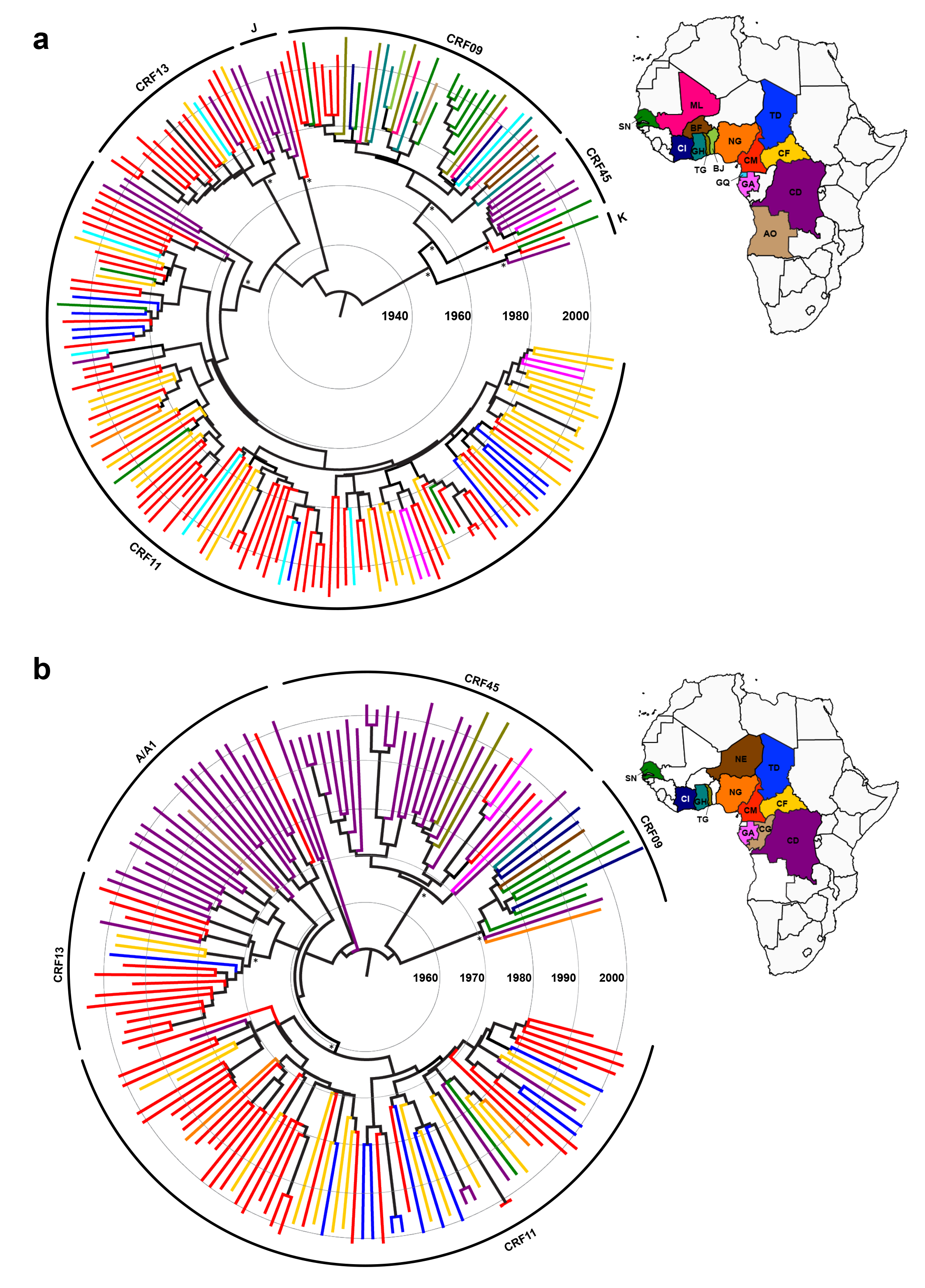

Supplement: Additional file 7: — Figure S4. Geographic distribution of the HIV-1 CRFs09/11/13/45_cpx pol (a) and env (b) gene fragments. Tips colors indicate the country of isolation of each sequence, according to the map. Country names are indicated using a two-letter code in accordance with ISO 3166. The external circular segments highlight the position of each specific clade as indicated at the line. Asterisks point to key nodes with a high (>0.90) PP support. Branch lengths are drawn to scale with the concentric circles indicating years. The trees were automatically rooted under the assumption of a relaxed molecular clock. (TIF 2346 kb) [file 12862_2016_824_MOESM7_ESM.tif]
