# Supplementary material for: Time-scale of minor HIV-1 complex circulating recombinant forms from Central and West Africa
Source: BMC Evol Biol. 2016 Nov 16;16:249. doi: 10.1186/s12862-016-0824-8 (PMC5112642; doi:10.1186/s12862-016-0824-8)
Supplement: Additional file 4: — Table S1. Sequences reclassified in this study. (PDF 75 kb) [file 12862_2016_824_MOESM4_ESM.pdf]

Table S1. Sequences reclassified in this study

Accession Number	Previous classification	New classification	Gene
JQ796149	CRF11_cpx	CRF13_cpx	*pol*
AJ287004	J	CRF11_cpx	*pol*
AY665509	CRF09_cpx	CRF11_cpx	*env*
AF067758	A	CRF11_cpx	*env*
AF509519	A	CRF11_cpx	*env*
AJ272643	A	CRF11_cpx	*env*
AJ389773	A	CRF09_cpx	*env*
AJ404173	A	CRF45_cpx	*env*
AJ404201	A	CRF45_cpx	*env*
AJ630566	A	CRF45_cpx	*env*
AJ877647	A	CRF45_cpx	*env*
AJ877648	A	CRF45_cpx	*env*
AJ877653	A	CRF45_cpx	*env*
AJ877703	A	CRF45_cpx	*env*
AJ877871	A	CRF45_cpx	*env*
AJ877882	A	CRF45_cpx	*env*
AJ877904	A	CRF45_cpx	*env*
AM086523	A	CRF45_cpx	*env*
AM086546	A	CRF13_cpx	*env*
AY180113	A	CRF11_cpx	*env*
AY372235	A	CRF11_cpx	*env*
AY372236	A	CRF11_cpx	*env*
AY675600	A	CRF45_cpx	*env*
AY675605	A	CRF45_cpx	*env*
AY924577	A	CRF45_cpx	*env*
AY924578	A	CRF45_cpx	*env*
AY924597	A	A2	*env*
FN392867	A	CRF45_cpx	*env*
U43136	A	CRF11_cpx	*env*
U43139	A	CRF11_cpx	*env*
U69994	A	CRF11_cpx	*env*
U69996	A	CRF11_cpx	*env*
X80453	A	CRF11_cpx	*env*
X80454	A	CRF11_cpx	*env*
AF028319	A1	CRF11_cpx	*env*
AF028324	A1	CRF11_cpx	*env*
AF028327	A1	CRF11_cpx	*env*
AF028328	A1	CRF11_cpx	*env*
AF509508	A1	CRF45_cpx	*env*

AF509522	A1	A2	*env*
AJ404011	A1	CRF11_cpx	*env*
AJ404035	A1	CRF45_cpx	*env*
AJ404047	A1	CRF45_cpx	*env*
AJ404070	A1	CRF09_cpx	*env*
AJ404081	A1	CRF11_cpx	*env*
AJ404133	A1	CRF45_cpx	*env*
AJ490714	A1	CRF13_cpx	*env*
AJ490723	A1	CRF11_cpx	*env*
AJ490731	A1	CRF11_cpx	*env*
AJ490766	A1	CRF11_cpx	*env*
AJ490768	A1	CRF11_cpx	*env*
AJ554719	A1	CRF13_cpx	*env*
AJ554850	A1	CRF11_cpx	*env*
AJ554856	A1	CRF11_cpx	*env*
AJ554901	A1	CRF13_cpx	*env*
AJ554954	A1	CRF11_cpx	*env*
AJ554955	A1	CRF11_cpx	*env*
AM903398	A1	CRF45_cpx	*env*
FM955823	A1	CRF45_cpx	*env*
FM955836	A1	CRF45_cpx	*env*
KC200478	A1	CRF09_cpx	*env*
AF028316	A2	A1	*env*
AF028323	A2	A1	*env*
AF028325	A2	A1	*env*
AF028331	A2	A1	*env*
AJ404024	A2	CRF45_cpx	*env*
EU031851	A2	A1	*env*
