# Supplementary material for: Time-scale of minor HIV-1 complex circulating recombinant forms from Central and West Africa
Source: BMC Evol Biol. 2016 Nov 16;16:249. doi: 10.1186/s12862-016-0824-8 (PMC5112642; doi:10.1186/s12862-016-0824-8)
Supplement: Additional file 5: — Table S2. HIV-1 CRFs_cpx pol dataset. (PDF 69 kb) [file 12862_2016_824_MOESM5_ESM.pdf]

Table S2. HIV-1 CRFs_cpx *pol* dataset.

CRF/Subtype	Country	*N*[*]	Sampling date
CRF09_cpx	AO	1	2001
	BF	2	2004-2006
	BJ	1	2008
	CI	2	2000-2001
	CM	6	1999-2010
	GH	5	1996-2009
	GQ	1	2008
	ML	4	2006
	SN	10	1995-2009
	TG	4	2009-2011
CRF11_cpx	CD	4	2002-2007
	CF	32	1999-2009
	CM	57	1995-2010
	GA	4	2000-2008
	GQ	5	2006-2008
	NG	1	2010
	SN	4 (1)	1998-2011
	TD	9	2006-2007
CRF13_cpx	CD	3	2002-2007
	CF	2	2005-2009
	CM	14 (1)	1996-2009
	GQ	1	2003
CRF45_cpx	CD	6	1997-2007
	CM	1	1997
	GA	1	1997
	SN	1	2009
J	CD	4	1993-2002
	CM	1	2004
K	CD	1	1997
	CM	1	1996
	SN	1	2009

* The numbers in parenthesis represent the sequences reclassified in this work as depicted in Table S1.
