# Supplementary material for: Time-scale of minor HIV-1 complex circulating recombinant forms from Central and West Africa
Source: BMC Evol Biol. 2016 Nov 16;16:249. doi: 10.1186/s12862-016-0824-8 (PMC5112642; doi:10.1186/s12862-016-0824-8)
Supplement: Additional file 6: — Table S3. HIV-1 CRFs_cpx env dataset. (PDF 70 kb) [file 12862_2016_824_MOESM6_ESM.pdf]

Table S3. HIV-1 CRFs_cpx *env* dataset.

CRF/Subtype	Country	*N*[*]	Sampling date
CRF09_cpx	CD	1 (1)	1997
	CI	3 (1)	2000-2009
	GH	1	1996
	NE	1	2000
	NG	1 (1)	1996
	SN	5	1994-2007
CRF11_cpx	CD	5 (3)	1984-1997
	CF	14 (3)	1990-1999
	CM	34 (16)	1993-2002
	NG	2	1994-1996
	SN	1 (1)	1997
	TD	11 (4)	1999-2000
CRF13_cpx	CD	2 (1)	2002-2003
	CF	2	1998
	CM	12 (2)	1994-2004
	TD	1 (1)	1999
CRF45_cpx	CD	22 (19)	1997-2004
	CM	2 (1)	1997-2000
	GA	3 (2)	1997-2004
	TG	2 (2)	2006
A/A1	AO	32 (1)	2001
	CG	11	1996-1997
	CD	245	1985-2003
	CF	7	1995-1998
	CI	53	1997-2009
	CM	162 (4)	1993-2001
	GA	10	1988-2004
	GH	3	1990-2002
	GW	4	2005-2006
	ML	3	2003-2004
	NE	1	2000
	NG	6	1996
	SN	9	1996-2007
	TD	15	1999-2000
	TG	6	2006
A2	AO	2 (1)	2001
	CD	5	1997-2002
	CM	2 (1)	2000-2001
	NE	1	2000

* The numbers in parenthesis represent the sequences reclassified in this work, as depicted in Table S1.
