# Supplementary material for: Time-scale of minor HIV-1 complex circulating recombinant forms from Central and West Africa
Source: BMC Evol Biol. 2016 Nov 16;16:249. doi: 10.1186/s12862-016-0824-8 (PMC5112642; doi:10.1186/s12862-016-0824-8)
Supplement: Additional file 8: — Table S4. Best-fit demographic models for HIV-1 CRF11_cpx pol and env datasets. (PDF 64 kb) [file 12862_2016_824_MOESM8_ESM.pdf]

Table S4. Best-fit demographic models for HIV-1 CRF11_cpx *pol* and *env* datasets.

Gene	Model	PS Log ML	Models compared	Log BF	SS Log ML	Models compared	Log BF
pol	Log	**-16343.8**	-	-	**-16343.8**	-	-
	Expo	-16366.0	Log/Expo	22.2	-16366.2	Log/Expo	22.4
	Expa	-16376.8	Log/Expa	33.0	-16376.8	Log/Expa	33.0
env	Log	-7471.3	-	-	-7471.3	-	-
	Expo	-7475.6	Log/Expo	4.3	-7475.5	Log/Expo	4.2
	Expa	-7537.6	Log/Expa	66.3	-7537.6	Log/Expa	66.3

Log marginal likelihood (ML) estimates for the logistic (Log), exponential (Expo) and expansion (Expa) growth demographic models obtained using the path sampling (PS) and stepping-stone sampling (SS) methods. The Log Bayes factor (BF) is the difference of the Log ML between of alternative (H1) and null (H0) models (H1/H0). Log BFs > 3 indicates that model H1 is more strongly supported by the data than model H0.
